# Health Inequalities amongst People of African Descent in the Americas, 2005–2017: A Systematic Review of the Literature

**DOI:** 10.3390/ijerph16183302

**Published:** 2019-09-08

**Authors:** Sandra del Pino, Sol Beatriz Sánchez-Montoya, José Milton Guzmán, Oscar J. Mújica, Juan Gómez-Salgado, Carlos Ruiz-Frutos

**Affiliations:** 1 Cultural Diversity, Office of Equity, Gender and Cultural Diversity, Pan American Health Organization, PAHO/WHO, Washington, DC 20037, USA; 2 Independent Consultant, Bogota 235367, Colombia; 3 Health and Cultural Diversity, El Chaco Region, Pan American Health Organization, PAHO/WHO, Asuncion 595-21, Paraguay; 4 Social Epidemiology and Health Equity, Department of Evidence and Intelligence for Action in Health, Pan American Health Organization, PAHO/WHO, Washington, DC 20037, USA; 5 Department of Sociology, Social Work and Public Health, University of Huelva, 21007 Huelva, Spain; 6 Safety and Health Posgrade Program, Universidad Espíritu Santo, Guayaquil 091650, Ecuador

**Keywords:** systematic review, social determinants of health, health inequalities, social inequity, ethnicity, African-American

## Abstract

Ethnic inequalities are often associated with social determinants of health. This study seeks to identify the latest scientific evidence on inequalities in the health of people of African descent in the Americas. For this, a systematic review of the literature on health and people of African descent in the Americas was carried out in the LILACS, PubMed, MEDLINE, and IBECS databases. Institutional and academic repositories were also consulted. Evidence was obtained on the presence and persistence of health inequalities in the population of African descent in the Americas from the identification of five types of quantitative and qualitative evidence: (1) ethnic/racial concept and variables; (2) relations with other social determinants; (3) health risks; (4) barriers and inequalities in health services; and, (5) morbi-mortality from chronic diseases. Studies with qualitative methods revealed invisibility, stereotypes, and rejection or exclusion as main factors of inequality. This review evidenced the existence of health inequalities, its interconnection with other adverse social determinants and risk factors, and its generation and perpetuation by discrimination, marginalization, and social disadvantage. These conditions make people of African descent a priority population group for action on equity, as demanded by the 2030 Agenda for Sustainable Development.

## 1. Introduction

International organizations, such as the United Nations, use resolutions to promote a focus on ethnic/racial and intercultural equality [1]. The purpose of this paper is to identify evidence on inequalities in the health of people of African descent, that can support decision making for health policies, strategies, and action plans to help overcome ethnic and racial inequality in the region of the Americas.

The search was based on the definition of “Afro-descendant” used as a reference for the PAHO/WHO regional Policy on Ethnicity and Health [2], adopted in 2017 by the Member States of the Pan American Health Organization: “In Latin America and the Caribbean, this refers to the different black or Afro-American cultures that emerged from the descendants of Africans who survived the transatlantic human trafficking, or slave trade, that flourished from the 16th to the 19th century”. This definition served as the starting point for an exploration of search strategies.

The concepts of ethnicity and race are those considered in the 2017 Economic Commission for Latin America and the Caribbean (ECLAC) report Situación de las personas afrodescendientes en América Latina y desafíos de políticas para las garantías de sus derechos ]. The concepts of race and ethnicity are not synonymous and are, undeniably, social constructs. However, ethnic identity has greater depth and stability than racial identity because it is not based solely on phenotypic characteristics and their meanings, but also relates to a set of collective attributes shared by an ethnic community and from one generation to another [3]. This publication stresses that inequalities based on ethnic and racial attributes are not simply remnants of a colonial past, but rather constitute contemporary mechanisms that reproduce existing frameworks and produce new frameworks through which discriminated people are kept in a position of disadvantage [3]. This study considers the cultural racism approach described by Lee, such as the assumption of the ideology, values, behaviors and norms of the dominant group as the identifying characteristics of society, excluding the non-dominant group [4].

Ethnicity seems to play an important role in prenatal care as identified by Green. It has been associated with unequal perinatal counseling regarding diet, smoking, drinking, medication use, breastfeeding, baby development and early labor [5]. Also, immigrants from southern Africa are more likely to experience higher lifetime child mortality compared to the native-born population [6].

Racial health inequalities have been previously studied in America. A research conducted aiming at comparing racial inequalities in health in the United States and Canada revealed that black–white and Hispanic–white inequalities were relatively larger in the United States, while aboriginal–white inequalities were larger in Canada [7].

The present study, as an exploratory review of the literature, set out to find quantitative and qualitative evidence of health inequalities among people of African descent and other population groups in the Americas. As a secondary objective, it sought to identify publications that could contribute to a better understanding of the mechanisms that generate inequalities in health and living conditions between populations of African descent and those not of African descent [8].

## 2. Materials and Methods

### 2.1. Design

This study aims at specifically exploring the public health literature on inequalities amongst people of African descent in the Americas. The search strategy did not include other specialized databases (such as sociology, economics, or anthropology).

The following research question was proposed: What public health evidence exists about health inequalities related to the (historical, social, and cultural) ethnic conditions of people of African descent in the Americas, compared to other population groups?

This question was used to design two search queries, one in Spanish using *Descriptores en Ciencias de la Salud* (DeCS) and one in English using Medical Subject Headings (MeSHs). These queries were supplemented by a search of the institutional repositories of several universities (Antioquia, Nariño, Los Andes, and Rosario in Chile; University ICESI in Colombia; the Autonomous University of Mexico), the Latin American Council of Social Sciences- Comparative Research Programme on Poverty CLACSO-CROP in Latin America and the Caribbean; ECLAC; the Ministries of Health of Colombia, Mexico, Brazil, and the Ministry of Culture of Colombia; and the National Urban League (NUL) (See Table 1).

### 2.2. Eligibility Criteria

The eligibility criteria for the publications considered for this review were:

#### Inclusion Criteria

(1) People of African descent in the Americas as the population of interest.
(2) Published from 2005 to 2017.
(3) Written in English, Portuguese, or Spanish.
(4) For quantitative studies: report or demonstrate the use of methodologies and measurement instruments to establish an association between African descent and social inequalities in health.
(5) For qualitative studies: use methodologies and assessment instruments to establish an association between African descent and social inequalities in health.
(6) Contextual framework considers historical, social, and cultural factors that affect the living conditions of people of African descent in the Americas.
(7) Research approach considers how inequalities and inequities in health are generated among people of African descent in the Americas.
(8) Address differences in equality, equity in health, or disease (morbidity and mortality) outcomes between people of African descent in the Americas and other population groups.
(9) Consider factors that generate cumulative effects in terms of ethnic inequalities and inequities among people of African descent in the Americas (e.g., female gender, living in remote rural areas).

**Figure 1:**
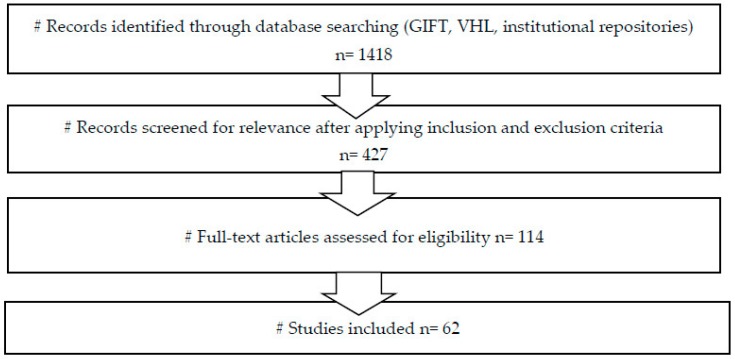
Flow diagram of evidence selection from primary studies.

The initial search of WHO Global Information Full Text (GIFT), Virtual Health Library (VHL), and the aforementioned institutional repositories retrieved 1418 records. After screening of titles and abstracts and application of the inclusion and exclusion criteria, 427 records remained. These were selected for full-text reading and assessment of eligibility, based on the extent of their contribution to understanding the situation of health inequalities among people of African descent and those not of African descent in the Americas. After this step, 114 articles remained. After a final round of review and consultation with experts on health inequalities and ethnicity, 62 articles were selected (See Figure 1).

The theoretical framework of this review was the model developed by Weightman [9] for the evaluation of public health interventions. This model is particularly useful for systematic reviews because it allows the evaluation of non-analytical studies with different designs, as well as expert opinions. The Preferred Reporting Items for Systematic Reviews and Meta-Analyses (PRISMA) statement was followed throughout.

### 2.3. Operating Concepts

#### 2.3.1. Quantitative Research

Studies that work on sequential, deductive and probative processes; that measure, use statistics, analyze causal relationships, and generate results for purposes of generalization, replication, or prediction [10].

#### 2.3.2. Qualitative Research

Studies that use inductive processes to analyze subjective reality; rather than measuring or using statistics, they work out ideas in depth, address the understanding, interpretation, and meanings of data, and contextualize phenomena. Their results are not generalizable [10].

## 3. Results

Ultimately, 62 studies conducted between 2005 and 2017 were included in the review. Of the 64 records identified in the LILACS, MEDLINE, PubMed, and IBECS databases through the GIFT/VHL portal, 20 were retrieved from GIFT, 16 from the VHL, 1 from Equity Health, and 25 from institutional repositories. Of the selected publications, 42 were scholarly articles, 18 were documents, and two were presented as educational material for virtual study.

From the 62 selected articles, 32 were carried out using qualitative methods and 30 with quantitative methods (See Table 2).

Of the 62 publications, six (9.67%) were reviews (desk review, state-of-the-art, or literature review); only one was a systematic review (See Table 3).

Three broad types of quantitative evidence were identified: one regarding inequalities in negative health outcomes; one obtained from data such as censuses and surveys; and one obtained from the use of statistical tools to measure marginalization and segregation (See Table 4).

The most relevant quantitative research identified within the aforementioned typology is presented in Table 5.

Six broad types of qualitative evidence were identified in the present review: historical evidence related to the legal/political recognition, design, implementation, and evaluation of health policies targeting people of African descent in settings of social and institutional discrimination; on the public health consequences of inequalities; on how the concepts of ethnicity and race are used in data collection instruments; on representations, behaviors, and various forms of racism; on the territorial, political, and cultural organization of people of African descent; and on traditional medicine and midwifery. (See Table 6).

The most relevant qualitative research identified within the aforementioned typology is presented in Table 7.

## 4. Discussion

The findings of this review show how complex this topic is. In terms of time, there is a tendency to make the health inequality situation for people of African descent more visible as well as based on a more scientific quantitative evidence. Regarding the subjects addressed, there is also a tendency to highlight epidemiological and clinical studies, particularly on non-communicable diseases and external causes. Notwithstanding this tendency, the focus seems to have shift towards analyzing the problem from a social sciences perspective to provide a greater explanatory capacity. Our findings also pinpoint the challenge of conducting studies with mixed methods that will allow a better understanding of the mechanisms that generate, transmit, and perpetuate health inequalities.

The results of this review contribute to the construction of an evidence-based framework to support decision making about health policies, programs, plans, and technical protocols with the aim of eliminating or reducing health inequalities in people of African descent, in a manner consistent with the Sustainable Development Goals (SDGs) pledge of “leaving no one behind” [8].

Quantitative research has fundamentally focused on the social determinants of health, risk factors, and negative or positive health outcomes. Some authors have stressed the need for more research into the mechanisms whereby poverty, social injustice, and ethnic and cultural factors act as barriers to contribute to the generation and perpetuation of inequalities, especially as they pertain to negative outcomes [11]. These mechanisms may include discriminatory forms of access to high-quality health services [12]. Some authors have mentioned the need to address ethnicity in an intersectional manner with other variables, such as social class and gender [13].

Some authors have focused on addressing ethnic disparities for access and use of health services in different situations and in different outcomes. These studies show how people of African descent are at a disadvantage to access prevention services, care, hospitalizations, use of innovative therapies or high technology in health (See Table 8).

Several studies also recognized the importance of improving the quality of data, particularly by including ethnicity/race as a variable in censuses, surveys, and continuous records [14,15,16]. Others have proposed the application of quantitative methods to conduct georeferencing of racial segregation and discrimination [17,18].

Qualitative research, in turn, allows us to move forward with some reflections and begin other, necessary discussions. Advances include the availability of international instruments, which can be administered differently in different geographical areas and recognize people of African descent as subjects collectively deserving of the same social, economic, and cultural rights. This allows progress in the understanding and application of instruments to eliminate racism and discrimination.

Qualitative studies also provide a better understanding of the positive impact of taking a cultural approach to the organization of territories and communities of African descent, based on their ethnic and racial identities. To do so, some authors have proposed the concept of “place and effect” [19]. Other studies addressed territorial approaches to the practice of traditional medicine and midwifery in Afro-descendant communities [20]. Similar settings produced evidence on social and community strategies to fight discrimination [21].

Some papers analyzed social and occupational conditions as determinants of health, assigning particular importance to ethnic inequalities in labor mobility [22] and the migration of Haitians [23].

The Brazilian scientific community has made significant contributions regarding health policies for the Black population, generating elements to further current understanding of how to design, implement, develop indicators for, monitor, and evaluate such policies from an ethnic perspective.

There is a particular need for expanded use of mixed methods, which combine quantitative research to identify the dimension and severity of inequalities and qualitative approaches to understand why and through which mechanisms these inequalities occur. An example of such an approach is provided by a study on ethnic inequalities and high-risk behaviors in HIV [24].

There is a need to address the challenge of adopting an intercultural approach within the context of the social determinants of health. By considering the gender and ethnic inequalities that interact with one another, the differences in access to health throughout the life course, as well as the promotion and respect of individual rights and, in the case of indigenous peoples, collective rights [73], this particular need can be covered.

Our study has certain limitations. The most relevant one, perhaps, is the scope of the search strategy being restricted to the public health area. However, the results of our exploratory review point to the need to expand this search to broader social sciences areas such as sociology, economics, anthropology, and the like. Finally, we identified an unmet need for a research protocol to support systematic reviews of health issues—such as the present one—which do not conform to the requirements traditionally used for systematic reviews of mostly clinical topics.

## 5. Conclusions

To conclude, this review evidenced the existence of health inequalities associated to the ethnic-racial status of the Afro-descendant populations in the Americas. The findings of this review show the complexity of this topic and highlight the importance of the social sciences perspective to gain greater explanatory capacity. The interconnection between ethnic-related health inequalities with other adverse social determinants (e.g., territorial spatial segregation, poor living conditions, social and institutional exclusion, poverty, migration) and risk factors (e.g., informal mining, exposure to chemicals and urban pollutants, poor basic sanitation) generate intersectional inequalities that perpetuate discrimination, marginalization, and social disadvantages. These conditions make people of African descent a priority population group for action on equity, as demanded by the 2030 Agenda for Sustainable Development.

## Figures and Tables

**Table 1:** Descriptores en Ciencias de la Salud (DeCS) and Medical Subject Headings (MeSHs) descriptors.

Spanish *	English **
“Determinantes Sociales en Salud” AND Afro$ “Determinantes Sociales en Salud” AND etnicidad “Determinantes Sociales en Salud” AND “étnico-racial” “Determinantes Sociales en Salud” AND “grupos étnicos” “Disparidades Sociales en Salud” AND Afro$ “Disparidades Sociales en Salud” AND etnicidad “Disparidades Sociales en Salud” AND “étnico-racial” “Disparidades Sociales en Salud” AND “grupos étnicos” “Revisión sistemática exploratoria”	“Social Determinants of Health” AND “African Continental Ancestry Group” “African Continental Ancestry Group” AND “Health Inequalities” “African Continental Ancestry Group” AND “Social Inequity” “Systematic review” AND “African Continental Ancestry Group”

Source: Own work. * The Spanish search query was run in the Virtual Health Library (VHL) and institutional repositories. ** The English search query was run in the WHO Global Information Full Text (GIFT) project.

**Table 2:** Characteristics of the publications included in the systematic exploratory review on health inequalities and people of African descent.

Ref. Num.	Country, Year of Publication	Title of Document	Type of Publication	Authors	Type of Evidence
[11]	USA, 2008	Social Determinants of Black–White Disparities in Breast Cancer Mortality: A Review	Scholarly article	Gerend, M.A.; Manacy, P.	Quantitative
[12]	USA, 2010	Increased Black–White disparities in mortality after the introduction of lifesaving innovations: a possible consequence of US federal laws	Scholarly article	Levine, R.S.; Rust, G.S.; Pisu M.; et al.	Quantitative
[13]	Mexico, 2010	La inequidad por clase, etnia y género expresada en el desmedro	Scholarly article	González, R.; García, J.J.; Tinoco, A.	Qualitative
[14]	Chile, 2005	Pueblos indígenas y afrodescendientes de América Latina y el Caribe: información sociodemográfica para políticas y programas	Document	Fabiana-Del Popolo, M.A.	Qualitative
[15]	Chile, 2009	Afrodescendientes en América Latina y el Caribe: Del reconocimiento estadístico a la realización de derechos	Scholarly article	Anton, J.; Bello, A.; Fabiana-Del Popolo, M.A.; Paixao, M.	Qualitative
[16]	Brazil, 2016	A panorama of health inequalities in Brazil	Document	Landmann-Szwarcwald, C.; Macinko, J.	Quantitative
[17]	USA, 2008	Resilience to urban poverty: Theoretical and empirical considerations for population health	Scholarly article	Sanders, A.E.; Lim, S.; Sohn, W.	Quantitative
[18]	Colombia, 2015	Desigualdades sociales, ¿Inequidades espaciales? Análisis de la segregación sociorracial en Bogotá (2005–2011)	Scholarly article	Villamizar-Santamaría, S.	Quantitative
[19]	Mexico, 2007	De las “tres razas” al mestizaje: diversidad de las representaciones colectivas acerca de lo “negro” en México (Veracruz y Costa Chica)	Scholarly article	Hoffmann, O.	Qualitative
[20]	Colombia, 2013	Rescatar la medicina tradicional en la salud materno infantil de comunidades afrocaucanas a través de diálogo intercultural	Scholarly article	Portela Guarín, H.; Astaiza, N.; Guerrero, N.; Rodríguez, S.	Qualitative
[21]	Mexico, 2017	Construcción del sujeto de derecho afrodescendiente en México. Reflexiones desde el Pacífico Sur mexicano	Scholarly article	Lara Millán, G	Qualitative
[22]	Colombia, 2008	Desigualdad socioracial frente a la movilidad laboral en Cali	Scholarly article	Olivier Barbary; Alexander Estacio Moreno	Qualitative
[23]	Chile, 2015	Racismo y matrices de “inclusión” de la migración haitiana en Chile: elementos conceptuales y contextuales	Scholarly article	Rojas, N.; Amode, N.; Vásquez, J.	Qualitative
[24]	Canada, 2013	HIV risk perception and distribution of HIV risk among African, Caribbean and other black people in a Canadian city	Scholarly article	Baidoobonso, S.; Bauer, G.R.; Speechley, K.N.	Quantitative
[25]	USA, 2009	Addressing Social Determinants of Health to improve access to Early Breast Cancer Detection: Results of the Boston REACH 2010 Breast and Cervical Cancer Coalition Women’s Health Demonstration Project	Scholarly article	Clark, C.R.B.; Nashira Kunicki, M.C.	Quantitative
[26]	USA, 2010	Validation of the group-based medical mistrust scale among urban black men	Scholarly article	Shelton, R.C.; Winkel, G.; Davis, S.N.; et al.	Quantitative
[27]	Hawaii, 2010	Mortality patterns of native Hawaiians across their lifespan: 1990–2000	Scholarly article	Panapasa, S.V.; Mau, M.K.; Williams, D.R.; et al.	Quantitative
[28]	USA, 2011	HIV prevalence rates among men who have sex with men in the southern united states: population-based estimates by race/ethnicity	Scholarly article	Lieb, S.; Prejean, J.; Thompson, D.R.; et al.	Quantitative
[29]	USA, 2011	Place, Not race: Disparities Dissipate in Southwest Baltimore When Blacks and Whites Live Under Similar Conditions	Scholarly article	Laveist, T.; Pollak, K.; Thorpe, R.	Quantitative
[30]	USA, 2011	Despite Improved Quality of Care in The Veterans Affairs Health System, Racial Disparity Persists for Important Clinical Outcomes	Scholarly article	Trivedi, A.N.; Grebla, R.C.; Wright, S.M.	Quantitative
[31]	USA, 2011	Temporal trends in the black/white breast cancer case ratio for estrogen receptor status: disparities are historically contingent, not innate	Scholarly article	Krieger, N.; Jarvis T.; Waterman P.D.	Quantitative
[32]	USA, 2012	Race differences in the physical and psychological impact of hypertension labeling	Scholarly article	Spruill, T.M.; Gerber, L.M.; Schwartz, J.E.; et al.	Quantitative
[33]	USA, 2012	Racial disparities in hematopoietic cell transplantation in the United States	Scholarly article	Majhail, N.S.; Nayyar, S.; Santibanez, M.E.B.	Quantitative
[34]	USA, 2013	Black women’s awareness of breast cancer disparity and perceptions of the causes of disparity	Scholarly article	Karen Kaiser, K.A.; Gina Curry, K.	Quantitative
[35]	USA, 2013	The effect of race, ethnicity, and mood/anxiety disorders on the chronic physical health conditions of men from a national sample	Scholarly article	Johnson-Lawrence, V.; Griffith, D.M.; Watkins, D.C.	Quantitative
[36]	Brazil, 2013	The role of potential mediators in racial inequalities in tooth loss: the Pró-Saúde study	Scholarly article	Celeste, R.K.; Goncalves, L.G.; Faerstein, E.; et al.	Quantitative
[37]	Brazil, 2014	Comparing adult users of public and private dental services in the state of Minas Gerais, Brazil	Scholarly article	Pinto, R.D.; de Abreu, M.H.N.G.; Vargas, A.M.D.	Quantitative
[38]	English-speaking Caribbean, 2015	Disparities in cardiovascular disease among Caribbean populations: a systematic literature review	Scholarly article	Francis, D.K.; Bennett, N.R.; Ferguson, T.S.	Quantitative
[39]	USA, 2015	HIV infection among people who inject drugs in the United States: geographically explained variance across racial and ethnic groups	Scholarly article	Linton, S.L.; Cooper, H.L.F.; Kelley, M.E.	Quantitative
[40]	Canada, 2016	The mental health status of ethnocultural minorities in Ontario and their mental health care	Scholarly article	Grace SL. Tan; RA Cribbie	Quantitative
[41]	Latin America, 2010	Análisis de Determinantes Sociales de la desnutrición en Latinoamérica	Scholarly article	Jiménez-Benítez; A. Rodríguez-Martín, R.	Qualitative
[42]	Mexico, 2011	Los determinantes sociales, las desigualdades en salud y las políticas, como temas de investigación	Scholarly article	Santos Padrón, H.	Qualitative
[43]	Brazil, 2012	Recorte étnico-racial	Scholarly article	Garcia de Pinto da Cunha, E.E.	Qualitative
[44]	Brazil, 2012	A equidade racial nas políticas de saúde	Scholarly article	Deivison Mendes, F.	Qualitative
[45]	Brazil, 2013	Iniquidades raciais e saúde: o ciclo da política de saúde da população negra	Scholarly article	LBatista, L.E.; Batista Monteiro, R.	Qualitative
[46]	Colombia, 2015	Inequidad en la utilización de servicios de salud reproductiva en Colombia en mujeres indígenas y afrodescendientes	Scholarly article	Noreña-Herrera, C.; Leyva-Flores, R.; Palacio-Mejía, L.S.; Duarte-Gómez, M.B.	Qualitative
[47]	Brazil, 2016	Educational inequalities in hypertension: complex patterns in intersections with gender and race in Brazil	Document	Fernandes, R.; Santos Alves, E.F.	Qualitative
[48]	Colombia, 2009	Derecho a la salud grupos étnicos en Bogotá	Scholarly article	González-Acosta, A.	Qualitative
[49]	Latin America, 2010	Mortalidad infantil en la niñez indígena y afrodescendiente de América Latina: inequidades estructurales, patrones diversos y evidencia de derechos no cumplidos	Document	ECLAC, UNFPA (United Nations Populations Fund), PAHO.	Qualitative
[50]	Colombia, 2015	Ambigüedades en dos décadas de paradigma multiculturalista. Algunos elementos de la historia inmediata de los Afrocolombianos.	Scholarly article	Valencia Angulo, L.E.	Qualitative
[51]	Mexico, 2006	Afrodescendientes en México: Reconocimiento y propuestas antidiscriminación	Document	CONAPRED (Consejo Nacional para prevenir la discriminación)	Qualitative
[52]	Nicaragua, 2007	Desigualdades sociodemográficas en Nicaragua: tendencias, relevancia y políticas pertinentes (Acuerdo de Cooperación CEPAL, Comisión Económica para América Latina y el Caribe,-UNFPA Oficina de Nicaragua)	Document	Delgadillo, M.	Qualitative
[53]	USA, 2007	Evolución del concepto etnia/raza y su impacto en la formulación de políticas para la equidad (OPS/OMS)	Self-learning material	Torres-Parodi, C., Bolis, M.	Qualitative
[54]	Colombia, 2008	Discriminación étnico-racial, desplazamiento y género en los procesos identitarios de la población “negra” en sectores populares de Bogotá	Scholarly article	Meertens, D., Viveros, M., Arango L.G.	Qualitative
[55]	Chile, 2008	Visibilidad estadística de la población afrodescendiente de América Latina: aspectos conceptuales y metodológicos	Document	Antón, J.; Del Popolo, F.	Qualitative
[56]	USA, 2009	Etnicidad y Salud	Self-learning material	Torres, C.; Burbano, E.	Qualitative
[57]	Chile, 2010	La variable etnia/raza en los estudios de estratificación social	Document	Sepúlveda Sánchez, D.	Qualitative
[58]	Cuba, 2010	Población Cubana ante factores de riesgos para la salud	Document	Alfonso León, A.C.	Qualitative
[59]	USA, 2011	La situación de las personas afrodescendientes en las américas	Document	OAS (Organization of American States)	Qualitative
[60]	Brazil, 2011	Saúde e comunidades quilombolas: Uma revisão da literatura	Document	Antunes Freitas, D.; Diaz Caballero, A.; et al.	Qualitative
[61]	Peru, 2011	El uso de categorías étnico/raciales en censos y encuestas en el Perú: balance y aportes para una discusión	Document	Valdivia Vargas, N.	Qualitative
[62]	Colombia, 2012	Equidad en la Diferencia: Políticas para la Movilidad Social de Grupos de Identidad Misión de Movilidad Social y Equidad	Document	Cárdenas, J.C.; Ñopo, H.; Castañeda, J.L.	Qualitative
[63]	Brazil, 2012	O recorte étnico-racial nos sistemas de informações em saúde do Brasil	Scholarly article	Soares Filho, A.M.	Qualitative
[64]	Brazil, 2012	O movimento negro na construção da política nacional de saúde integral da população negra e sua relação com o estado brasileiro. In: *Saúde da População Negra*	Scholarly article	Assis Brasil, S.; Alves Bomfi, L.	Qualitative
[65]	Colombia, 2013	Situación alimentaria y nutricional en Colombia desde la perspectiva de los determinantes sociales de la salud	Scholarly article	Álvarez Castaño, L.S.; Pérez Isaza, E.J.	Qualitative
[66]	Colombia, 2014	Plan especial de salvaguarda de los saberes asociados a la partería afro del pacífico	Document	Quiñones Sánchez, L.; López, G.; Valencia, T.; Cuero Valencia, S. Gómez Lozano, B.	Qualitative
[67]	Colombia, 2015	Observatorio para la medición de Desigualdades y Análisis de Equidad en Salud	Document	Colombian Ministry of Health and Social Protection.	Quantitative
[68]	Colombia, 2015	Estudios afrocolombianos en la antropología: tres décadas después	Scholarly article	Restrepo, E.	Qualitative
[69]	USA, 2015	State of Black America	Document	National Urban League (NUL).	Quantitative
[70]	Colombia, 2015	La interculturalidad: ¿principio o fin de la utopía?	Scholarly article	Castillo Guzmán, E.; Guido Guevara, S.P.	Qualitative
[71]	Mexico, 2015	Derechos colectivos y reconocimiento constitucional de las poblaciones afromexicanas	Document	Velázquez, M.E.; Iturralde Nieto, G.; Ramírez Caloca, S.	Qualitative
[72]	Mexico, 2017	Análisis espacial de la desigualdad social	Document	Argüelles Enríquez, E.; Uriel Lomelí, C.	Quantitative

**Table 3:** Review articles included.

Ref. Num.	Author	Country	Study Design	Study Contribution
[11]	Gerend, M.A. et al., 2008	USA	Literature review	Ethnic inequalities in cancer.
[20]	Portela Guarín, H. et al., 2013	Colombia	Desk review	The *diálogo de saberes* technique as a mechanism to understand inequality.
[38]	Francis, D.K. et al., 2015	English-speaking Caribbean	Systematic review	Cardiovascular disease stratified by ethnicity.
[42]	Santos Padrón, H. 2011	Mexico	Literature review	Ethnicity/race as a conditioning factor of health inequalities.
[43]	Garcia de Pinto da Cunha, E. 2012	Brazil	State-of-the-art review	Use of ethnicity/race variables in health to improve visibility and data quality.
[61]	Antunes Freitas, D. 2011	Brazil	State-of-the-art review	Black rural communities.

Source: Own work.

**Table 4:** Relevant quantitative studies and their contributions to understanding health inequalities among people of African descent in the Americas.

Ref. Num.	Author	Country	Study Design	Level of Evidence	Study Contribution
[13]	González-Guzmán, R. et al., 2010	Mexico	Retrospective cohort study.	Robust	Triple inequality: “Class, ethnicity, and gender”.
[14]	Del Popolo, F. (Ed.) 2005	Chile	Descriptive studies (compilation).	Moderate	Statistical information on people of African descent and use of the ethnicity variable in censuses.
[15]	Anton, J. et al., 2009	Chile	Descriptive studies (compilation).	Robust	Statistical visibility of people of African descent.
[16]	Landmann-Szwarcwald, C. et al., 2016	Brazil	Special issue of the *International Journal for Equity in Health* about inequities in Brazil.	Robust	Nationwide health surveys as a source of data for inequality research.
[17]	Sanders, A.E. et al., 2008	USA	Longitudinal social-epidemiological study.	Robust	Resilient environments as a protective factor.
[20]	Villamizar-Santamaría, S.F. 2015	Colombia	Estimation of dissimilarity indices and location coefficients for spatial analysis of racial segregation.	Robust	Ethnic/racial inequalities as expressed by spatial segregation rates.
[24]	Baidoobonso, S. et al., 2013	Canada	Mixed-methods study.	Robust	Ethnic inequalities and high-risk behaviors in HIV.
[25]	Clark, C. et al., 2009	USA	Prospective cohort study.	Robust	Quality of health care provided to African-American women with cancer.
[29]	Laveist, T. et al., 2011	USA	Comparative study of black and white persons exposed to equal living conditions.	Robust	Ethnic inequalities in health under similar socioeconomic conditions.
[30]	Trivedi, A.N. et al., 2011	USA	Evaluation of organizational changes in health services.	Robust	Ethnic inequalities and non-communicable diseases.
[33]	Majhail, N.S. et al., 2012	USA	Review of the association between access to hematopoietic cell transplantation and ethnicity.	Robust	Ethnic inequalities in timely access to hematopoietic cell transplantation.
[35]	Johnson-Lawrence, V. et al., 2013	USA	Covariance study of mood, chronic conditions, and ethnicity.	Robust	Ethnic inequalities, anxiety disorders, and chronic disease.
[36]	Celeste, R.K. et al., 2013	Brazil	Longitudinal cross-sectional study on ethnic inequalities.	Robust	Ethnic inequalities and social determinants of oral health.
[39]	Linton, S.L. et al., 2015	USA	Variance and georeferencing study.	Robust	Ethnic disparities in HIV and injectable drug use.
[40]	Grace, S.L. et al., 2016	Canada	Cross-sectional analytical study on the relationship between access to mental health services and ethnicity.	Robust	Ethnic-cultural differences, psychosocial distress, and access to mental health services.
[67]	Colombian Ministry of Health and Social Protection. 2015	Colombia	Methodological guideline for measurement of inequalities.	Robust	Methodologies for measurement of ethnic inequalities.
[72]	Argüelles Enríquez, E. et al., 2017	Mexico	Estimation of marginalization rates and spatial correlations thereof.	Robust	Marginalization rates, spatial correlation, and people of African descent.

Source: Own work.

**Table 5:** Quantitative studies included in this review, stratified by type of evidence provided.

Box: Number and Percentage of Quantitative Studies Identified.		
Type of Evidence	n	%
Studies on inequalities as they pertain to risk factors and negative health and/or nutrition outcomes between different ethnic groups.	19	30.64
Nationwide health surveys, studies, or tools designed to collect data with a view to addressing ethnic inequalities in social determinants and/or health systems and services.	9	14.52
Quantitative evidence on georeferencing, spatial autocorrelation, marginalization indices, dissimilarity indices, location coefficients, and measures of segregation.	2	3.22
TOTAL	30	48.38

Source: Own work.

**Table 6:** Quantitative studies included in this review, stratified by type of evidence provided.

Box: Number and percentage of qualitative studies identified		
Type of Evidence	n	%
Historical evidence of the contrast between advances in political-legal recognition and structural and institutional discrimination, racial inequalities in health, and racism as a socio-historical phenomenon.	10	16.14
Evidence of racial inequalities in the health status of the population as a public health issue.	8	12.90
Evidence on the concepts used in censuses, surveys, and studies to define management of ethnic/racial variables.	7	11.29
Evidence on the ways in which ethnic and ethnic-territorial representations and stereotypes with effects on social behaviors, such as racism and sexist racism, are generated and reproduced.	3	4.84
Qualitative evidence on elements which define people as being of African descent, such as physical characteristics, identity, social and territorial sense of belonging, cultural traditions, historicity, organizational processes, and religious myths.	2	3.22
Evidence on traditional medicine and midwifery among people of African descent.	2	3.22
TOTAL	33	51.61

Source: Own work.

**Table 7:** Relevant qualitative studies and their contributions to understanding health inequalities among people of African descent in the Americas.

Ref. Num.	Author	Country	Study Design	Quality of Evidence	Study Contribution
[19]	Hoffmann, O. 2007	Mexico	Qualitative study on racial identities at the local level.	Moderate	Ethnic-racial identities and the concept of “place and effect”.
[21]	Lara Millán, G. 2017	Mexico	Characterization of the Afro-Mexican population on the basis of territorial, identity-related, and cultural variables.	Moderate	Social and community strategies against discrimination.
[22]	Barbary, O. et al., 2008	Colombia	Survey on career paths and ethnicity.	Robust	Social and labor-related ethnic inequalities as they pertain to labor mobility.
[23]	Rojas Pedemonte, N. et al., 2015.	Chile	Qualitative ethnographic study.	Limited	Migration of Afro-Haitians.
[34]	Kaiser, K. et al., 2013	USA	Survey and focus groups with Black women.	Robust	Inequalities in cancer mortality among Black women.
[41]	Jiménez-Benítez, A. et al., 2010.	Latin America	Analysis of the social and ethnic dimensions of malnutrition.	Limited	Participatory public policies and ethnicity in nutritional health.
[44]	Faustino DM. 2012	Brazil	Analysis of the implementation of public policies for people of African descent.	Robust	Evaluation of public policies pertaining to health and ethnicity.
[45]	Batista, L.E. et al., 2013.	Brazil	Analysis of the implementation of public policies for people of African descent.	Moderate	Implementation of health-related public policies and the Black population.
[46]	Flores Dávila, J.I. 2006	Mexico	Qualitative mixed-methods study.	Robust	Afro-descendant territories and communities.
[48]	González-Acosta, A. 2009	Colombia	Analysis of public policies in ethnicity and health.	Robust	Public policies, human rights, and ethnic groups.
[53]	Torres-Parodi, C. et al., 2007	USA	Periodization of international instruments.	Limited	International instruments against racism, discrimination and inequality.
[56]	Torres-Parodi, C. et al., 2009	USA	Analysis of ideologies and social representations about racism.	Limited	African-Americans as historical and collective subjects.
[58]	Alfonso León, A.C. 2010	Cuba	Qualitative study on risk factors and skin color.	Moderate	Risk factors in health and cultural behaviors.
[59]	OAS, 2011	USA	Questionnaire sent to Member States and civil society organizations.	Moderate	Social, economic, and cultural rights of people of African descent.
[62]	Cárdenas, J.C. 2012	Colombia	Census review to identify the statistical visibility of the Afro-Colombian population.	Robust	Statistical invisibility in health and African descent.
[63]	Martins Soares Filho, A. 2012	Brazil	Risk and protective factors for chronic non-communicable diseases.	Moderate	Availability of racial data and ethnic/racial inequalities.
[64]	Assis BrasilAlves Bomfi, L. 2012	Brazil	Desk review.	Moderate	Public health policies for the Black population.
[66]	Quiñones Sánchez, L. et al., 2014	Colombia	Interviews with focus groups and traditional midwives.	Limited	Traditional medicine and midwifery in Afro-descendant communities.

Source: Own work.

**Table 8:** Studies that address ethnic disparities on access and use of health services.

Ref. Num.	Autor	Country	Study Design	Study Contribution
[11]	Gerend, M.A. et al., 2008.	USA	Literature review.	Need to address cultural barriers and people of African descent.
[12]	Levine, R.S. et al., 2010.	USA	Descriptive study.	People of African descent accessing to innovative therapies for HIV treatment.
[16]	Landmann-Szwarcwald, C. et al., 2016.	Brasil	Evaluation of Health policies and programs.	Equity in Access to health services.
[25]	Clark, C. et al., 2009.	USA	Prospective cohort.	Access to continuous care for timely follow-up of Afro women diagnosed with breast cancer.
[30]	Trivedi, A.N. et al., 2011	USA	Evaluative study of health services.	Effects of the transformation of health services for war veterans among people of African descent and the rest.
[33]	Majhail, N.S. et al., 2012	USA	Literature review	Access to hematopoietic cell transplantation by people of African descent.
[34]	Kaiser, K. et al., 2013.	USA	Qualitative study on perceptions.	Access to cancer prevention and control services by women of African descent.
[37]	Pinto, R.D. et al., 2014	BRA	Cross-sectional.	Type of oral health services used by people of African descent.
[38]	Francis, D.K. et al., 2015.	English Caribbean	Systematic literature review.	Ethnic disparities in the use of health services and hospital admission.
[40]	Grace, S.L.; et. Al. 2016.	Canada	Service evaluation study.	Ethnic disparities in the use of mental health services.
[42]	Santos Padrón, H. 2011	Mexico	Documentary review.	Ethnic disparities in health services.
[46]	Noreña-Herrera, C. et al., 2015.	Colombia	Regression study.	Use of reproductive health services in indigenous and afro-descendant women.
